# Editorial: What can we make of theories of embodiment and the role of the human mirror neuron system?

**DOI:** 10.3389/fnhum.2015.00500

**Published:** 2015-09-11

**Authors:** Analía Arévalo, Juliana Baldo, Fernando González-Perilli, Agustín Ibáñez

**Affiliations:** ^1^Center for Aphasia and Related Disorders, East Bay Institute for Research and EducationMartinez, CA, USA; ^2^Center for Basic Research in Psychology and Faculty of Information and Communication, University of the RepublicMontevideo, Uruguay; ^3^Department of Basic, Evolutionary and Educational Psychology, Universitat Autonoma de BarcelonaBarcelona, Spain; ^4^Laboratory of Experimental Psychology and Neuroscience, Institute of Cognitive Neurology (INECO), Favaloro UniversityBuenos Aires, Argentina; ^5^National Scientific and Technical Research Council (CONICET)Buenos Aires, Argentina; ^6^UDP-INECO Foundation Core on Neuroscience, Diego Portales UniversitySantiago, Chile; ^7^Department of Psychology, Universidad Autónoma del CaribeBarranquilla, Colombia; ^8^Centre of Excellence in Cognition and its DisordersSydney, NSW, Australia

**Keywords:** human mirror system, embodiment, grounded cognition, mirror neurons, language processing

Over the last 20 years, work surrounding theories of embodiment and the role of the putative mirror neuron system (MNS) in humans has been hotly debated. In 2000, Ramachandran (2000, p. 1) suggested that mirror neurons would do for psychology what DNA did for biology, providing “a unifying framework” that would help explain a host of mental abilities.” In fact, the strong evidence for action/perception coupling observed in macaque mirror neurons led several authors to implicate this system in higher order functions in humans, such as empathy, language and theory of mind (Rizzolatti and Arbib, 1998; Gallese et al., 2004; but see Hickok, 2009). Thus, embodiment is a broad area of study that suggests that motor resonance participates in several of these higher order processes. However, the exact role played by specific brain structures and/or actual mirror neurons in these processes varies greatly across theories and authors. This special issue brought together 12 studies conducted with healthy as well as brain-injured populations, behavioral as well as imaging techniques (functional and structural), and opinion pieces and responses. Through this broad landscape, we offer a fresh and frugal approach to the challenges and controversies of the translational neuroscience of embodiment and the MNS.

Two of the articles in this collection addressed how the human MNS might underlie the physiological mechanisms that give rise to human emotions. In “Motor empathy is a consequence of misattribution of sensory information in observers,” Mahayana et al. (2014) used TMS to measure participants' reactions while they observed videos of painful stimuli being inflicted on another person. Their results suggest that empathy may be partially caused by a misattribution of perceptual information: pain experienced in someone else is perceived as occurring in oneself. This finding raises an interesting and novel view on embodiment that suggests that the empathy experienced through our mirror system is in fact selfish, as it mostly reflects empathy toward ourselves. In “Washing the guilt away: effects of personal versus vicarious cleansing on guilty feelings and prosociality,” Xu et al. (2014) asked participants to write about a guilt-inducing past wrong and were then asked to wash their hands, watch a video of someone washing their hands, or a video of someone typing. They were then asked whether they would help a Ph.D. student with her thesis by answering some questions. Participants who felt the least guilty were those who washed their hands, followed by those who watched the hands-washing video, and then by those who watched the typing video. Also, participants who felt most guilty were more likely to help the student with her project. The authors conclude that washing one's hands or watching someone else washing their hands can be good for feelings of guilt, but not compassion. Both studies offer new evidence for the connection between inner ‘motor resonance’ and emotion (i.e., Wicker et al., 2003). Also, the study by Xu et al. and that of Kacinik (see below) are classic examples of embodied language, where even the enactment of metaphorical expressions can strongly activate the mirror neuron system.

In “Language comprehension warps the mirror neuron system,” Zarr et al. (2013) asked participants to read sentences describing the transfer of objects away or toward the reader. The adapting sentences disrupted prediction of actions in the same direction, but (a) only for videos of biological motion, and (b) only when the effector implied by the language (e.g., the hand) matched the videos. Similarly, Kacinik (2014) asked participants to read a story and act out the idioms presented (e.g., literally sitting on the fence, on the edge of one's seat) in “Sticking your neck out and burying the hatchet: what idioms reveal about embodied simulation.” They found that the process of embodying idioms simply by engaging in the corresponding actions activated their meaning enough to significantly influence subsequent processing and judgments. Finally, in “Action relevance in linguistic context drives word-induced motor activity,” Aravena et al. (2014) analyzed online modulations of grip force while subjects listened to target words embedded in different linguistic contexts. They conclude that motor structure activation is part of a dynamic process that integrates the lexical meaning potential of a term and the context in the online construction of a situation model, which is a crucial process for fluent and efficient online language comprehension. Similarly to Xu et al. (see above), these three articles support the notion that the motor resonance of language strongly influences its comprehension. The strict version of this view, which argues that semiotic coding would mostly rely on the human MNS (see Pulvermüller et al., 2014), continues to be controversial and is challenged by other articles in this topic (see below).

Two articles used neuroimaging to identify the neural correlates of embodiment. In an fMRI study entitled “Hand specific representations in language comprehension,” Moody-Triantis et al. (2014) asked participants to perform right or left hand actions and then read sentences describing these same actions. They found that language-induced activity overlapped with pre-motor and parietal regions associated with action planning rather than those observed in action execution, endorsing a less strict interpretation of the MNS in humans, in which association (and not primary motor cortices) are activated. In “Neuroanatomical substrates of action perception and understanding: an anatomic likelihood estimation meta-analysis of lesion-symptom mapping studies in brain injured patients” (2014), Urgesi et al. (2014) conducted a meta-analysis of 11 studies and 361 patients and reported that non-linguistic action perception and understanding are associated with the inferior frontal cortex, the inferior parietal cortex and the middle/superior temporal cortex. Again, rather than primary motor cortex, they found that surrounding regions in frontal, parietal, and temporal cortex were associated with action perception.

Two other theoretical/opinion articles also steer away from stricter MNS interpretations and suggest that the motor system influences action perception but is not its sole critical component. In “Homuncular mirrors: misunderstanding causality in embodied cognition,” Mikulan et al. (2015) propose a network view of language processing in which the mirror neuron system plays an important role in priming or facilitating understanding (or even indexing action semantics) but not directly in action understanding. Similarly, Bach et al. (2014) propose an object-based view of action understanding in “The affordance-matching hypothesis: how objects guide action understanding and prediction.” They suggest that object knowledge (what an object is for and how it is used) informs and constrains action interpretation and prediction.

Additionally, we included two response pieces to Bach et al.'s proposal, one by Osiurak (2014) and the other by Uithol and Maranesi (2014). The latter, in turn, received a response from Bach and colleagues (under review), which is also included in this issue. Osiurak proposes the “mechanical knowledge hypothesis,” which diminishes the role of manipulation in action understanding and distances itself from traditional MN theories, while Uithol and Maranesi support an enactivist view, which criticizes the need for integrating the processes of action interpretation and action prediction. On the other hand, Bach et al.'s counter argument suggests that the match is indeed needed to fulfill the requirements of a predictive model of action understanding.

Intriguingly, in “Observation and imitation of actions performed by humans, androids and robots: an EMG study,” Hofree et al. (2015) show that these phenomena are not limited to agents with a biological appearance but also for robotic agents, opening important implications regarding human-robot interaction.

All of these works expand our understanding of the human MNS by extending previous work and delimiting the boundaries of how we should interpret those findings. As a group, contributing authors seem to agree on less strict interpretations of embodiment and the human MNS, suggesting these are strong contributors to various aspects of action and cognition, but do not represent the sole basis of language, learning, or comprehension. Future work should further explore the precise mechanisms underlying the links between action planning, execution, and semantic processing, as well as the relative dependence of distinct cognitive processes on mirror activity.

## Conflict of interest statement

The authors declare that the research was conducted in the absence of any commercial or financial relationships that could be construed as a potential conflict of interest.

## References

[B1] Aravena P. Courson M. Frak V. Cheylus A. Paulignan Y. Deprez V.. (2014). Action relevance in linguistic context drives word-induced motor activity. Front. Hum. Neurosci. 8:163. 10.3389/fnhum.2014.00163 24744714 PMC3978346

[B2] Bach P. Nicholson T. Hudson M. (2014). The affordance-matching hypothesis: how objects guide action understanding and prediction. Front. Hum. Neurosci. 8:254. 10.3389/fnhum.2014.00254 24860468 PMC4026748

[B3] Gallese V. Keysers C. Rizzolatti G. (2004). A unifying view of the basis of social cognition. Trends Cogn. Sci. 8, 396–403. 10.1016/j.tics.2004.07.002 15350240

[B4] Hickok G. (2009). Eight problems for the mirror neuron theory of action understanding in monkeys and humans. J. Cogn. Neurosci. 21, 1229–1243. 10.1162/jocn.2009.21189 19199415 PMC2773693

[B5] Hofree G. Urgen B. A. Winkielman P. Saygin A. P. (2015). Observation and imitation of actions performed by humans, androids and robots: an EMG study. Front. Hum. Neurosci. 9:364. 10.3389/fnhum.2015.00364 26150782 PMC4473002

[B6] Kacinik N. A. (2014). Sticking your neck out and burying the hatchet: what idioms reveal about embodied simulation. Front. Hum. Neurosci. 8:689. 10.3389/fnhum.2014.00689 25309381 PMC4173310

[B7] Mahayana I. T. Banissy M. Chen C. Y. Walsh V. C. H. Muggleton N. G. (2014). Motor empathy is a consequence of misattribution of sensory information in observers. Front. Hum. Neurosci. 8:47. 10.3389/fnhum.2014.00047 24567713 PMC3915178

[B8] Mikulan E. P. Reynaldo L. Ibáñez A. (2015). Homuncular mirrors: misunderstanding causality in embodied cognition. Front. Hum. Neurosci. 8:299. 10.3389/fnhum.2014.00299 24860479 PMC4026706

[B9] Moody-Triantis C. Humphreys G. F. Gennari S. P. (2014). Hand specific representations in language comprehension. Front. Hum. Neurosci. 8:360. 10.3389/fnhum.2014.00360 24917803 PMC4042095

[B10] Osiurak F. (2014). Mechanical knowledge, but not manipulation knowledge, might support action prediction. Front. Hum. Neurosci. 8:737. 10.3389/fnhum.2014.00737 25278867 PMC4166253

[B11] Pulvermüller F. Moseley R. L. Egorova N. Shebani Z. Boulenger V. (2014). Motor cognition-motor semantics: action perception theory of cognition and communication. Neuropsychologia 55, 71–84. 10.1016/j.neuropsychologia.2013.12.002 24333695

[B12] Ramachandran V. S. (2000). Mirror Neurons and Imitation Learning as the Driving Force Behind “The Great Leap Forward” in Human Evolution. Edge 69. Available online at: www.edge.org/documents/archive/edge69.html

[B13] Rizzolatti G. Arbib M. A. (1998). Language within our grasp. Trends Neurosci. 21, 188–194. 10.1016/S0166-2236(98)01260-0 9610880

[B14] Uithol S. Maranesi M. (2014). No need to match: a comment on Bach, Nicholson and Hudson's “Affordance-Matching Hypothesis.” Front. Hum. Neurosci. 8:710. 10.3389/fnhum.2014.00710 25309386 PMC4159984

[B15] Urgesi C. Candidi M. Avenanti A. (2014). Neuroanatomical substrates of action perception and understanding: an anatomic likelihood estimation meta-analysis of lesion-symptom mapping studies in brain injured patients. Front. Hum. Neurosci. 8:344. 10.3389/fnhum.2014.00344 24910603 PMC4039011

[B16] Wicker B. Keysers C. Plailly J. Royet J. P. Gallese V. Rizzolatti G. (2003). Both of us disgusted in My insula: the common neural basis of seeing and feeling disgust. Neuron 40, 655–664. 10.1016/S0896-6273(03)00679-2 14642287

[B17] Xu H. Bègue L. Bushman B. (2014). Washing the guilt away: effects of personal versus vicarious cleansing on guilty feelings and prosocial behavior. Front. Hum. Neurosci. 8:97. 10.3389/fnhum.2014.00097 24616686 PMC3937805

[B18] Zarr N. Ferguson R. Glenberg A. M. (2013). Language comprehension warps the mirror neuron system. Front. Hum. Neurosci. 7:870. 10.3389/fnhum.2013.00870 24381553 PMC3865370

